# IgMk paraprotein from gammopathy patient can bind to cardiolipin and interfere with coagulation assay: a case report

**DOI:** 10.1186/s12865-017-0213-0

**Published:** 2017-06-23

**Authors:** Xin-yao Wu, Yu-feng Yin, Jia-lin Teng, Li-wei Zhang, Cheng-de Yang

**Affiliations:** 1 Department of Rheumatology and Immunology, Ruijin Hospital, Shanghai Jiaotong University School of Medicine, No. 197 Ruijin Second Road, Huangpu District, Shanghai, 200025 China; 2 Department of Clinical Laboratory, Ruijin Hospital, Shanghai Jiaotong University School of Medicine, Shanghai, 200025 China

**Keywords:** Paraprotein, Monoclonal Gammopathy, Antibody, Phospholipid, Coagulation

## Abstract

**Background:**

The monoclonal gammopathies are a group of plasma-cell proliferative disorders characterized by the secretion of monoclonal immunoglobulin (M protein or paraprotein). Some rare cases have revealed the specific affinity of paraprotein as autoantibody. Here we report a patient with monoclonal gammopathy of undetermined significance (MGUS) accompanied by a remarkable increase of anticardiolipin antibody (aCL) and an extensively decreased coagulation factor activity, however, without any clinical signs of antiphospholipid syndrome (APS) and bleeding.

**Results:**

Our further investigation indicated that IgMκ paraprotein of this patient possessed an antibody activity against phospholipids so as to bind to cardiolipin and interfere with coagulation assay in vitro.

**Conclusions:**

This case might be indicative that an abnormality of coagulation tests, disturbed by IgMκ paraprotein, does not predict a risk of bleeding in this patient.

## Background

The monoclonal gammopathies, including monoclonal gammopathy of undetermined significance (MGUS) and multiple myeloma, are a group of plasma-cell proliferative disorders characterized by the secretion of monoclonal immunoglobulin [1]. MGUS denotes a preneoplastic entity that can develop into multiple myeloma or other lymphoproliferative disorders including macroglobulinemia or primary amyloidosis [2]. The incidence of MGUS, in a progression risk of 1% per year, is approximately 1 to 3.4% in the general population from 50 to 70 years old [2, 3].

Unlike CRAB features (hypercalcaemia, renal failure, anaemia, and bone lesions) of multiple myeloma, systemic manifestations presented in MGUS are rare and extremely varied. However, several reports described that MGUS patients had both thrombolytic and hemorrhagic complications including deep venous thrombosis, acquired von Willebrand disease, prothrombotic abnormalities and acquired hemophilia [4–6]. Previous study suggested that these disorders in MGUS may be due to the antibody activity of the particular monoclonal immunoglobulin [6]. The specific affinity of paraprotein for thrombin or platelet glycoprotein IIIa was previously reported in two cases with severe bleeding disorders [7, 8]. Here we described a MGUS patient with significantly increased level of anticardiolipin antibody (aCL) and decreased levels of coagulation factor activity, however, without any clinical manifestations of antiphospholipid syndrome (APS) and bleeding. We demonstrate here that the high positivity of aCL and reduced activity of coagulation factors might be the result of a specific immunologic reaction of IgMκ paraprotein with phospholipids.

## Case report

A 63-year-old man presented to our hospital in January 2016, with chronic watery diarrhea lasting for more than 6 months, three to five times per day, without any visible traces of blood. He reported no abdominal pain, tenesmus, fever, bone pain or suspicious manifestations of bleeding. He suffered from diabetes mellitus for more than 10 years with a good self-blood glucose monitoring and no family pedigree was reported. Physical examination on admission revealed malnutrition and mild anemia, with no lymphadenopathy or hepatosplenomegaly. Incomplete intestinal obstruction was showed by abdominal CT scan 2 months before, however, not detected by gastroscopy and colonoscopy at admission.

Complete blood count showed a hemoglobin level of 80 g/L, a platelet count of 256 × 10^9^/L, and a white blood cell count of 4.89 × 10^9^/L with normal differentiation. The patient had a decreased level of serum total protein (45 g/L; normal range 60–83 g/L), albumin (24 g/L; 35–55 g/L), IgG (503 mg/dl; 751–1560 mg/dl) and a significantly raised IgM (984 mg/dl; 46–304 mg/dl). Serum levels of IgA and IgE were in the normal range. The enzyme-linked immunosorbent assay (ELISA) (EUROIMMUN, Lübeck, Germany) was used to detect the serum level of Antiphospholipid antibody (APLA) including aCL and anti-β2GP1 antibodies. Serum level of aCL (IgM) was significantly elevated up to 69.8 MPL/ml (normal range <12 MPL/ml) while serum aCL (IgG) (<2 GPL/ml; <12 GPL/ml) and anti-β2GP1 antibodies (11.2 RU/ml; <20.0 RU/ml) were within normal limits. In our laboratory, the reference range was established as titers equal or exceeding 99% of healthy pregnant and non-pregnant woman for aCL (IgM) (12 MPL/ml), aCL (IgG) (12 GPL/ml) and anti-β2GP1 antibodies (20.0 RU/ml) respectively. Lupus anticoagulant was 2.13 (>2 was defined as strong activity). The serum level of M paraprotein was 2.7 g/L and was determined as IgMκ type in immunofixation electrophoresis. More importantly, he had an extremely prolonged activated partial thromboplastin time (APTT) (114.7 s; 27.2–41.0 s), slightly increased prothrombin time (PT) (23.0 s; 10.0–16.0 s) and D-dimer (1.04 mg/L; <0.55 mg/L) (Table 1), together with a remarkable deficiency of all coagulation factors activity (Table 2).

**Table 1:** Demographic and blood parameters of the patient

Characteristics	Value	Reference (unit)
Age	63	year
Hemoglobin	80	g/L
Platelet count	256	85–303 × 10^9^/L
White blood cell count	4.89	3.97–9.15 × 10^9^/L
Serum total protein	45	60–83 g/L
Albumin	24	35–55 g/L
IgG	503	751–1560 mg/dl
IgM	984	46–304 mg/dl
Anticardiolipin antibody (IgM)	69.8	<12 MPL/ml
Anticardiolipin antibody (IgG)	<2	<12 GPL/ml
Anti-β2GP1 antibody	11.2	<20.0 RU/ml
Lupus anticoagulant	2.13	<1.2
M paraprotein (IgMκ)	2.7	g/L
Activated partial thromboplastin time	114.7	27.2–41.0 s
Prothrombin time	23.0	10.0–16.0 s
D-dimer	1.04	<0.55 mg/L
Bone marrow (FCM)		
CD19^+^ cell	11.1	%
CD19^+^CD20^+^ cell	98.2	%
CD19^+^CD38^+^ cell	94.5	%
CD19^+^CD138^+^ cell	<0.1	%
CD19^+^cyt-Igk^+^ cell	94.2	%
CD19^+^cyt-Igλ^+^ cell	4.4	%

*Abbreviations*: *FCM* Flow Cytometry Method

**Table 2:** Comparison of coagulation factor activity in patient plasma and purified IgMκ paraprotein plus normal control plasma

Coagulation factor	NC (% of activity)	Patient (% of activity)	IgMκ + NC (% of activity)
Factor VIII	183.6	3.1	6.1
Factor IX	111.9	2.6	3.9
Factor XI	87.7	3.5	3.3
Factor XII	78.9	6.5	3.5
Factor II	102.5	15.0	20.5
Factor V	90.6	40.3	39.1
Factor VII	112.6	58.9	38.5
Factor X	98.4	20.2	25.3

The reference of all coagulation factors: 50–150%; NC = normal control plasma; IgM + NC = purified IgMκ paraprotein plus normal control plasma (final concentration of IgMκ 1.72 g/L)

## Results

We hypothesized that the IgMκ paraprotein of this patient possessed a specific affinity for phospholipid, which served as anticardiolipin antibodies and disturb coagulation detection via binding to platelet phospholipid substitute in vitro. Therefore, we conducted a series of in vitro experiments to verify our hypothesis. The serum IgMκ paraprotein of this patient was purified using HiTrap IgM Purification HP and HiTrap Protein L columns (GE Healthcare, CA, USA) and the purity (>95%) was verified by SPIFE Immunofixation electrophoresis (IFE) Kits (HELENA Laboratories, Beaumont, TX, USA). Then, the IgMk sample was prepared by adding the purified IgMκ paraprotein to the normal control plasma and the final concentration was 1.72 g/L. Anticardiolipin antibody properties of IgMκ paraprotein was evaluated using aforementioned Anti-Cardiolipin kit (EUROIMMUN, Lübeck, Germany), and the result demonstrated a strong positivity (32.4 MPL/ml). On the other hand, the anticardiolipin activity of the purified IgM collected from a patient with Waldenström’s macroglobulinemia (WM), with a final concentration of 2.0 g/L after addition to the normal control plasma, was only 1.47 MPL/ml.

Finally, we repeated the coagulation tests on IL coagulation systems (HemosIL, MA, USA), our results showed that there was a significantly decrease of coagulation factors activity (VIII, IX, XI, XII, II, V, VII, and X) in our IgMk sample, indicating the inhibiting effects of IgMκ paraprotein on the assay of coagulation factors activity, the results were summarized in Table 2.

After ruling out the diagnosis of multiple myeloma and other B-cell proliferative disorder based on the evaluation of bone marrow biopsy, a diagnosis of MGUS was finally established. The patient was administrated by Clostridium butyricum and Pancreatin for diarrhea. His diarrhea disappeared with the aid of aforementioned symptomatic therapy for 10 days, but the abnormality in coagulation tests and the elevated M protein in electrophoresis of serum protein remained unchanged. In consideration of no further clinical manifestations, especially intestinal or hematological disorders, a regular follow-up was required for this patient.

## Discussion

We present here a patient with positive aCL antibodies and significantly reduced coagulation factor activity. Our experiments demonstrated an antibody property of IgMκ paraprotein that can bind to cardiolipin and interfere with assay of coagulation in this patient with MGUS. To explain the co-existence of positivity of aCL, hypocoagulability indicated by prolongation of APTT, the decrease in coagulation factors activity, and the absence of hemorrhagic disorders in clinical manifestation, we put forward a possible mechanism that the paraprotein might attack the phospholipid, a substitute of the platelet factor 3 which play an indispensable role in the common pathway of coagulation process in vivo [9]. Our results indicate that aCL positivity and the atypical coagulation detection might be a false positivity due to the specific antibody activity of IgMκ paraprotein against the phospholipid substitute in vitro.

Paraprotein have been thought of having antibody properties against clotting factors in several reported case [7, 10, 11]. A patient with similar prolongation of APTT and abnormality of multiple coagulation factors was reported to have severe bleeding and monoclonal antithrombin antibody [7]. Another case of Waldenstroem’s disease presented a high titer of monoclonal antiphospholipid antibodies, which did apparently not induce the clinical symptoms of antiphospholipid syndrome (APS) [10]. A third patient with IgMλ paraprotein and prolongation of APTT and PT, despite absence of bleeding manifestations and specific coagulation factor inhibition [11]. Above-mentioned cases shared both similarities and differences with our case. The mechanism of interaction between phospholipid and coagulation process as well as the production of monoclonal immunoglobulin remained complicated and unclear.

Besides specific affinity for autoantigens participated in coagulation process, antibody activities of monoclonal immunoglobulin reported in cases were highly varied, like IgM acting as anti-myelin-associated glycoprotein (MAG) antibodies in MGUS associated with peripheral neuropathy [12], IgM and IgG against transferrin in MGUS associated with transferrin-immune complex disease (TICD) [13] and IgG as antibodies against the CUB1-2 domains of ADAMTS13 in a MGUS patient associated with thrombotic thrombocytopenic purpura [14]. The existence of monoclonal immunoglobulin with specific affinity could also be found in multiple myeloma [15].

This is the first report of IgMκ paraprotein as antibodies against phospholipids to bind to cardiolipin and interfere with coagulation assay. This case provide an explanation for the dissociation of aberrant laboratory examination and clinical manifestation. Coagulation detection does not always reflect the real coagulation status in vivo. Great attention should be paid to the choice of hemostatic therapy or anticoagulation therapy in patients with antiphospholipid antibodies and coagulation abnormalities.

## Abbreviations

APTT: Activated partial thromboplastin time
MGUS: Monoclonal gammopathy of undetermined significance
PT: Prothrombin time
